# Quality of life and sexual function after tension-free vaginal tape and polyacrylamide hydrogel injection for primary stress urinary incontinence: 3-year follow-up from a randomized clinical trial

**DOI:** 10.1007/s00192-023-05626-x

**Published:** 2023-09-06

**Authors:** Anna-Maija Itkonen Freitas, Camilla Isaksson, Päivi Rahkola-Soisalo, Maarit Mentula, Tomi S. Mikkola

**Affiliations:** Department of Obstetrics and Gynaecology, Helsinki University Hospital, PO BOX 140, 00029 HUS Helsinki, Finland

**Keywords:** Mesh sling, Bulking, Polyacrylamide hydrogel

## Abstract

**Introduction and hypothesis:**

To assess quality of life (QoL) and sexual function outcomes at 3 years after tension-free vaginal tape (TVT) and polyacrylamide hydrogel injection (PAHG) for stress urinary incontinence (SUI).

**Methods:**

In this randomized trial comparing TVT (*n* = 104) and PAHG (*n *= 108), we assessed changes in QoL and sexuality using the Urogenital Distress Inventory (UDI-6), Incontinence Impact Questionnaire, Short Form (IIQ-7), Pelvic Organ Prolapse/Urinary Incontinence Sexual Questionnaire (PISQ-12) and RAND-36 Item Health Survey (RAND-36) at baseline and at 3 years. This is a secondary analysis of a randomized, noninferiority trial comparing patient satisfaction after TVT and PAHG.

**Results:**

In both groups, incontinence-related QoL improved from the baseline (*p* < 0.00), except for difficulty emptying the bladder and pain/discomfort. Total scores of UDI-6 and IIIQ-7 were lower for TVT compared to PAHG (*p* < 0.00) indicating better QoL at 3 years. Urinary incontinence with sexual activity or fear of incontinence restricting sexual activity improved in both groups (*p* < 0.00), with higher scores for physical section subscale in PISQ-12 (*p* = 0.02) for TVT. Physical and social functioning (RAND-36) improved from the baseline in both groups (*p* < 0.01) with a better outcome in the TVT group for physical functioning (*p* = 0.00).

**Conclusions:**

Both TVT and PAHG improve QoL and sexual function in primary SUI with better incontinence and health-related QoL scores in the TVT group compared to the PAHG group at 3 years.

## Introduction

Stress urinary incontinence (SUI) is defined as the complaint involuntary loss of urine on effort of physical exertion or on sneezing or coughing [1]. SUI affects up to 46% of adult women [2], and urinary incontinence has a marked effect on quality of life (QoL) [3]. Women suffering from urinary incontinence also report more sexual dysfunction compared to general, healthy women without urinary symptoms [4].

Pelvic floor muscle training increases QoL [5], but it is often insufficient to treat SUI. Mid-urethral sling surgery with retropubic tension-free vaginal tape (TVT) is a surgical treatment for SUI with a high success rate [6]. It has shown to improve QoL [6], and improve sexual function [7, 8]. However, after the US Food and Drug Administration warning in 2011, concerns about long-term mesh complications such as mesh erosion and chronic pain have been raised [9]. This substantially decreased the use of mid-urethral slings [10].

Urethral injection therapy with polyacrylamide hydrogel (PAHG, Bulkamid*®*) is a minimally invasive and safe intervention for SUI treatment [11, 12]. There are only limited data about QoL or sexual function following bulking treatments [12]. Previous studies including patients with mixed urinary incontinence and previous anti-incontinence surgery show improved QoL [13–15], and improved sexual function [13].

Retropubic TVT is the most common surgical treatment for SUI, however, urethral injection therapy with PAHG is becoming more popular in clinical use. Thus, comparative data with PAHG and the gold standard treatment is needed. SUI is a QoL symptom with a potential effect on sexual function. Our hypothesis was that both treatments could potentially improve QoL and sexual function.

We report data from a randomized clinical trial comparing TVT and PAHG in women with primary SUI. In this study our aim was to assess QoL and sexual function outcomes 3 years after TVT or PAHG and compare the groups. We used both urinary incontinence and general health-related quality of life (HRQoL) questionnaires. In this randomized study, 3-year midterm data was originally planned to be evaluated, and long-term data with 5-year follow-up will eventually be analyzed.

## Materials and methods

We conducted a prospective, randomized, controlled, parallel-group, non-inferiority trial comparing TVT and PAHG for primary SUI at Helsinki University Hospital. We recruited patients referred from primary healthcare with positive cough stress test, insufficient response after pelvic floor muscle physiotherapy, and eligible for TVT operation. Pelvic floor muscle therapy (PFMT) is the first-line treatment according to Finnish national guidelines on urinary incontinence, and women who were referred to our clinic had been earlier supervised by a health care professional or physiotherapist for PFMT, and due to inadequate response were seeking for operative treatment. Recruitment took place between September 28, 2015 and March 1, 2017. Study inclusion criteria included SUI not responsive to conservative treatment, patient age greater than 18 years, no previous incontinence procedure, a positive cough stress test without urge-type leakage, PVR volume less than 100 ml, and bladder capacity greater than 300 ml. In addition to the clinically used cough stress test, we used a pad test to confirm SUI [16]. Patients gave written informed consent, and the study was approved by the Helsinki University Ethics Committee and registered in Clinical Trials, NCT 02538991. The TVT (TVT-Exact®, Gynecare, Ethicon, Johnson & Johnson, USA) was performed according to the original description [17]. PAHG injections were inserted into four sites at 1.5 cm from the vesico-urethral junction under endoscopic control. All procedures were done in an outpatient setting using local anesthesia. Altogether, nine urogynecologist with strong background in SUI surgery performed the operations. Follow-up visits took place at 3 months, 1 year, and 3 years after the primary intervention. As part of the study protocol, one more PAHG injection (an addition or top up) after the initial PAHG was offered if the patient was not satisfied. Also, women unsatisfied with the given treatment and with persisting SUI confirmed with positive cough stress test at any follow-up visit were allowed to choose an alternative treatment (TVT or PAHG).

The primary noninferiority outcome of our study was patient satisfaction with the treatment ≥ 80, as measured by a VAS from 0 (extremely unsatisfied) to 100 (extremely satisfied) [18, 19], and QoL and sexual function analyses presented in this manuscript were secondary outcomes. We assessed symptom distress and the impact of incontinence on daily life using the Urogenital Distress Inventory (UDI-6) and Incontinence Impact Questionnaire, Short Form (IIQ-7).A total score ranging from 0 to 100 and higher points indicating more distress. The Pelvic Organ Prolapse/Urinary Incontinence Sexual Questionnaire (PISQ-12) was used to evaluate changes in sexual function [20]. Total scores comprise of three subscales (behavior–emotional, physical and partner-related), and higher scores indicate better sexual function. To measure general HRQoL we used RAND-36 Item Health Survey (RAND-36), with higher scores indicating better HRQoL [21]. The questionnaire includes eight multi-item dimensions: general health, physical functioning, mental health, social functioning, vitality, pain, and physical and emotional role functioning.

The sample size was calculated for the primary outcome, estimating that patient satisfaction with the treatment result of ≥ 80 on VAS from 0–100 was a good outcome. Based on earlier studies, the sample size was calculated to test two portions for noninferiority, setting the patient satisfaction with the treatment 80% in the TVT group and 75% in the PAHG group, assuming 5% unit predominance for TVT group [22–24]. The significance level was set at 5%, power at 80%, and noninferiority limit (threshold) at 20%. A dropout rate of 10% was assumed and thus, 212 women were planned to be randomized (1:1). Due to simultaneous outpatient clinic recruitment, 223 patients were randomized before closing the study using a computer-assisted random block system and R ((https://www.r-project.org/). This was done by an assistant outside the study. Randomization cards were sealed in opaque, sequentially numbered envelopes, and opened by the recruiting physician together with a nurse after the patient had signed the consent form. This trial was open label, in that participants or investigators were not masked to the treatment.

We analyzed data using IBM SPSS Statistics, version 24.0 (SPSS Inc., Chicago IL, USA). We present data from the questionnaires as means ± SD. We used the Student’s *t*-test for independent samples to analyze possible differences between the groups at 3 years. Changes in outcome measures within the groups before and 3 years after the treatment were analyzed using the Student’s *t*-test for paired samples. We present data in an intention-to-treat basis, but in order to confirm no significant difference due to crossover, we also analyzed the data as treatment received, including women who received only the treatment for which they were originally randomized. A *p*-value < 0.05 was considered significant for all analyses.

## Results

We randomized 223 women suffering from primary SUI to receive TVT (*n * = 110) or PAHG (*n* = 113) treatment at Helsinki University Hospital, Finland, as described before [18, 19]. Briefly, 212 women (TVT *n * = 104 and PAHG *n * = 108) underwent treatment as randomized, and we consider this to be the final sample size. Ninety-four women in the TVT group and 96 women in the PAHG group completed the 3-year follow-up questionnaires adequately and were included in this analysis. At 3 years in the TVT group, three women (3.2%) had received PAHG after TVT and no woman received a second TVT. In the PAHG group, 31 women (32.3%) had received only one PAHG injection, 35 women (36.5%) had received two PAHG injections, and 30 women (31.3%) had crossed over to receive TVT.

Baseline characteristics did not differ between the groups (Table 1). The mean BMI of the women was 25.0, the mean age was 51.0 years, and 43.4% were postmenopausal.

**Table 1:** Demographics of the 212 women undergoing TVT or PAHG treatment. Intention-to-treat data*

	TVT group *n* = 104	PAHG group *n* = 108
Age, mean, ± SD (range)	50.4 ± 10.4 (32.0–78.0)	51.5 ± 11.0 (31.0–80.0)
Postmenopausal	44 (42.3)	48 (44.4)
BMI (kg/m^2^), mean ± SD (range)	24.5 ± 3,5 (16.1–34.9)	24.8 ± 3.6 (18.9–34.2)
Smoking	14 (13.5)	10 (9.3)
Socioeconomical status		
Working	83 (79.8)	86 (79.6)
White collar workers	73 (70.2)	68 (62.9)
Blue collar workers	12 (11.5)	19 (17.6)
Others	19 (18.3)	21(19.5)
Parity/delivery, mean ± SD (range)	2.1 ± 1.0 (0–5)	2.1 ± 0.9 (0–6)
0	7 (6.7)	4 (3.7)
1	16 (15.4)	17 (15.7)
2	54 (51.9)	59 (54.6)
> 3	27(26.0)	28 (25.9)
Vaginal deliveries	93 (89.4)	101 (93.5)
Caesarean section only	4 (3.8)	3 (2.8)
Previous pelvic surgery	44 (42.3)	49 (45.4)
Distress from incontinence (VAS 0–10)**, mean ± SD (range)	8.0 ± 1.4 (4–10)	8.1 ± 1.4 (4–10)
Sexually active	72 (69.2)	71 (65.7)

*TVT* tension-free vaginal tape, *PAHG* polyacrylamide hydrogel, *SD* standard deviation, *BMI* body mass index, *VAS* visual analogue scale

^*^Data are presented as number of patients (%) unless otherwise stated

^**^n = 1 in PAHG group data missing

**Fig. 1:**
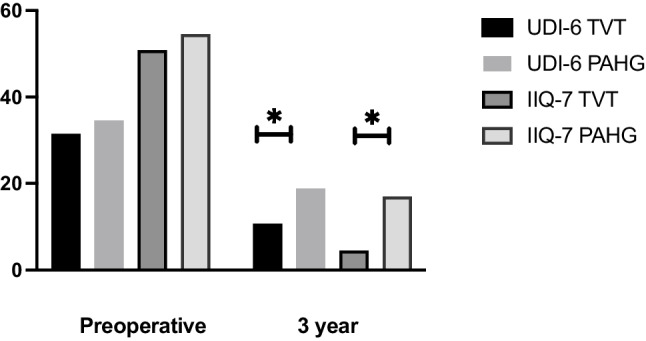
Mean scores for UDI-6 and IIQ-7, * = *p* < 0.001

The UDI-6 and IIQ-7 baseline scores did not differ between the groups, and highest distress was reported with stress leakage and impact of incontinence on physical activities (Tables 2, 3 and Fig. 1). In both groups, individual questions of UDI-6 and IIQ-7 showed significant improvement from the baseline except for difficulty emptying the bladder and pain or discomfort (Tables 2 and 3). Difficulty emptying bladder worsened in PAHG group from baseline (*p* = 0.00) but there was no difference between the groups at 3 years (Table 2). At 3 years, the TVT group had lower total scores compared to PAHG in UDI-6 (*p *< 0.00) and IIQ-7 (*p * < 0.00), indicating less urinary symptom related distress. There was no difference between the groups in frequent urination, difficulty emptying the bladder, and pain or discomfort, but all other individual UDI-6 and IIQ-7 questions also showed less distress for TVT patients at 3 years (Tables 2, 3).

**Table 2:** Results from UDI-6

UDI-6	Baseline TVT *n* = 104	Baseline PAHG *n* = 108	3 year TVT *n* = 94, *p* within-group difference	3 year PAHG n = 96, *p* within-group difference	*P* between-group difference
Frequent urination	0.99 ± 0.92	1.26 ± 0.94	0.57 ± 0.71, *p* ≤ 0.001 (difference 0.43, 95% CI 0.27–0.57)	0.74 ± 0.90, *p* ≤ 0.001 (difference 0.54, 95% CI 0.30–0.78)	0.16 (difference −0.17, 95% CI −0.40–0.07)
Urine leakage urgency	1.01 ± 0.96	1.23 ± 1.04	0.40 ± 0.66, *p* ≤ 0.001 (difference 0.59, 95% CI 0.39–0.80)	0.72 ± 0.80, *p* ≤ 0.001 (difference 0.60, 95% CI 0.38–0.83)	0.00 (difference −0.32, 95% CI −0.53—0.11)
Urine leakage stress	2.76 ± 0.47	2.78 ± 0.46	0.32 ± 0.59 , *p* ≤ 0.001 (difference 2.43, 95% CI 2.26–2.60)	1.10 ± 0.93, *p* ≤ 0.001 (difference 1.65, 95% CI 1.44–1.85)	< 0.001 (difference −0.78, 95% CI −1.01—0.56)
Dropping	2.06 ± 0.90	2.13 ± 0.78	0.54 ± 0.70, *p* ≤ 0.001 (difference 1.51, 95% CI 1.29–1.73)	0.97 ± 0.80, *p* ≤ 0.001 (difference 1.14, 95% CI 0.94–1.34)	< 0.001 (difference −0.42, 95% CI −0.64—0.21)
Difficulty emptying bladder	0.42 ± 0.68	0.43 ± 0.72	0.55 ± 0.67, *p* = 0.07 (difference -0.16, 95% CI -0.34–0.01*)*	0.71 ± 0.82, *p* = 0.002 (difference -0.27, 95% CI -0.09—3.12)	0.16 (difference −0.15, 95% CI −0.37–0.06)
Pain or discomfort	0.32 ± 0.63	0.39 ± 0.60	0.27 ± 0.64, *p* = 0.30 (difference 0.07, 95% CI -0.07–0.22)	0.29 ± 0.52, *p* = 0.29 (difference 0.06, 95% CI -0.06–0.18)	0.76 (difference −0.03, 95% CI −0.19–0.14)
Total score (max 100)	31.48 ± 11.51	34.57 ± 10.85	10.74 ± 9.88, *p* ≤ 0.001 (difference 20.74, 95% CI 18.20–23.27)	18.75 ± 13.40, *p* ≤ 0.001 (difference 15.66, 95% CI 12.44–18.88)	< 0.001 (difference −8.01, 95% CI −11.39—4.64)

**Table 3:** Results from IIQ-7

IIQ-7	Baseline TVT *n* = 104	Baseline PAHG *n* = 108	3 year TVT *n* = 94, *p* within-group difference	3 year PAHG *n* = 96, *p* within-group difference	*P* between- group difference
Household chores	1.12 ± 0.80	1.10 ± 0.90	0.04 ± 0.20, *p* ≤ 0.001 (difference 1.06, 95% CI 0.89–1.24)	0.28 ± 0.54, *p* ≤ 0.001 (difference 0.85, 95% CI 0.67–1.04)	< 0.001 (difference −0.24, 95% CI −0.36—0.13)
Physical acitivities	2.68 ± 0.56	2.68 ± 0.58	0.31 ± 0.06, *p* ≤ 0.001 (difference 2.39, 95% CI 2.24–2.54)	1.02 ± 0.94, *p* ≤ 0.001 (difference 1.63, 95% CI 1.41–1.84)	< 0.001 (difference −0.71, 95% CI −0.93—0.49)
Entertainment activities	1.56 ± 1.03	1.57 ± 0.87	0.09 ± 0.28, *p* ≤ 0.001 (difference 1.49, 95% CI 1.27–1.71)	0.41 ± 0.69, *p* ≤ 0.001 (difference 1.15, 95% CI 0.95–1.35)	< 0.001 (difference −0.33, 95% CI −0.48—0.18)
Ability to travel	1.17 ± 0.80	1.40 ± 1.04	0.15 ± 0.53, *p* ≤ 0.001 (difference 1.02, 95% CI 0.83–1.21)	0.38 ± 0.76, *p* ≤ 0.001 (difference 1.02, 95% CI 0.78–1.26)	0.02 (difference −0.23, 95% CI −0.42—0.04)
Participating social activities	1.34 ± 0.93	1.38 ± 0.86	0.11 ± 0.35, *p* ≤ 0.001 (difference 1.22, 95% CI 1.03–1.40)	0.39 ± 0.65, *p* ≤ 0.001 (difference 0.97, 95% CI 0.78–1.15)	< 0.001 (difference −0.27, 95% CI −0.42—0.12)
Emotional health	0.89 ± 0.90	1.17 ± 0.97	0.09 ± 0.28, *p* ≤ 0.001 (difference 0.81, 95% CI 0.62–0.99)	0.29 ± 0.63, *p* ≤ 0.001 (difference 0.83, 95% CI 0.64–1.03)	0.00 (difference −0.21, 95% CI −0.35—0.07)
Feeling frustrated	1.91 ± 0.92	2.11 ± 0.88	0.20 ± 0.52, *p* ≤ 0.001 (difference 1.76, 95% CI 1.56–1.96)	0.79 ± 0.90, *p* ≤ 0.001 (difference 1.26, 95% CI 1.04–1.49)	< 0.001 (difference −0.59, 95% CI −0.80—0.38)
Total score (max 100)	50.79 ± 19.01	54.50 ± 20.05	4.46 ± 8.87, *p* ≤ 0.001 (difference 46.72, 95% CI 42.62–50.81)	16.91 ± 19.56, *p* ≤ 0.001 (difference 36.71, 95% CI 31.75–41.68)	< 0.001 (difference −12.44, 95% CI −16.81—8.08)

At the baseline, 72 women (69.2%) in the TVT group and 71 women (65.7%) in the PAHG group were sexually active and at 3 years, 66 (70.2%) and 58 (60.4%) respectively. There was no difference between the groups in women remaining sexually active/inactive or turning sexually active/inactive at 3 years. Of all women at 3 years 115 (60.5%) remained sexually active, 50 (26.3%) remained sexually inactive, nine (4.7%) turned sexually active and 16 (8.4%) turned sexually inactive. The mean age of sexually inactive women was higher [61.0 ± 10.1 (40.0–38.0) vs 50.4 ± 8.7 (34.0–79.0)], and they were more often postmenopausal [52 (60.5%) vs 34 (39.5%)] compared to sexually active women. In both groups, questions 6 and 7 related to coital urinary incontinence (CUI) or fear of CUI restricting sexual activity showed significant improvement from the baseline at 3 years (*p* < 0.00) with higher scores in the TVT group compared to the PAHG group (*p *< 0.00 and *p* = 0.00 respectively). In the physical section subscale, significant improvement from the baseline was seen in both groups (*p* < 0.01), but TVT showed a better outcome at 3 years compared to PAHG (14.62 ± 1.68 vs 13.66 ± 2.75, difference 0.97, 95% CI 0.17–1.77). There was no difference between the groups in total scores at 3 years (36.94 ± 5.72 in the TVT-group vs 34.66 ± 7.87 in the PAHG group), although improvement from the baseline in total scores was only detected in the TVT-group (*p* < 0.00). No change in dyspareunia was detected in either group between the baseline and 3 years (*p* = 0.35 for TVT and  *p* = 0.88 for PAHG) and there was no difference between the groups at 3 years (*p *= 0.86).

**Fig. 2:**
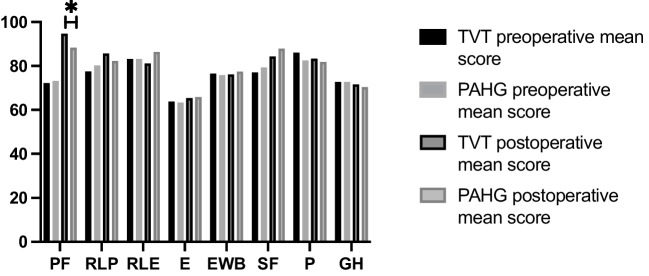
Mean scores for RAND-36, * = *p * < 0.001. *PF* = physical functioning, *RLP* = role limitations due to physical health, *RLE* = role limitations due to emotional problems, *E  *= energy/fatigue, *EWB* = emotional well-being, *SF* = social functioning, *P* = pain, *GH* = general health

RAND-36 showed significant improvement from the baseline in both groups in physical (*p* < 0.00) and social functioning (*p* = 0.01), with a better outcome in the TVT group compared to the PAHG group for physical functioning (*p* = 0.00, Fig. 2). Other dimensions of the questionnaire showed no difference between the groups.

## Discussion

Our results show that at 3 years both TVT and PAHG treatments improve incontinence-related QoL and sexual dysfunction and physical and social functioning of HRQoL. However, PAHG patients still suffered more from incontinence related distress at 3 years compared to the TVT group. This finding is in line with better objective and subjective efficacy of TVT compared to PAHG [19]. At 3 years intention-to-treat analysis, 95.7% of women in the TVT group and 78.1% in the PAHG group had negative cough stress test, and 100% and 88.5% respectively considered themselves subjectively cured or improved; 6.5% of women in the TVT group and 1.0% of women in the PAHG group reported tape/implantation site pain within the 3-year follow-up, and de-novo urgency occurred in 9.8% vs 12.5% of women respectively. Vaginal tape erosion requiring reoperation occurred in 3.3% of women in the TVT group [19]. Although occurring in small numbers, these complications might have an effect on QoL and sexual function.

In UDI-6 and IIQ-7, total scores showed lessened distress caused by urinary incontinence symptoms from the baseline in both groups, which is in line with previous studies after mid-urethral sling surgery [6] and less studied PAHG [25]. The improvement in QoL was also sustained from short-term [26] to 3 years. It has been shown that a total score above 25 in UDI-6 distinguishes between care and non-care seekers [27]. We show that in both our study groups a total score above 30 at the baseline was reduced after the treatments to below 20, indicating a satisfactory level at 3 years. Minimum important difference (MID) to assess clinically meaningful change in a questionnaire hasn’t been defined for UDI-6 and IIQ-7, but cutoff scores > 33.3 for UDI-6 and > 9.52 for IIQ-7 have been proposed to find individuals with symptomatic urinary incontinence [28]. Using these cutoff values in our data, both TVT and PAHG patients were asymptomatic at 3 years according to UDI-6. However, only the TVT group reached the cutoff score of IIQ-7, indicating the higher impact of urinary incontinence on QoL after treatment in the PAHG group.

Many studies have shown improved sexual function after mid-urethral sling surgery [7, 8, 29], but inconsistency between studies occurs [30]. Only one previous study has reported sexual function outcomes after PAHG treatment showing improvement in sexual function at 12 months [13]. In a recent study using the bulking agent polydimethylsiloxane Urolastic, sexual function also improved at 12 months after treatment [31]. Our study shows improvement in the physical subsection and incontinence-specific questions of PISQ-12 in both groups at 3-years. Although in total scores of PISQ-12 the improvement was seen for both groups at 12 months [26], at 3 years the total score improved from the baseline only in the TVT group. Sexual function is multifactorial, and the deterioration in behavior–emotional and partner-related subsections in the PAHG group is likely due to this. Also, no difference between the groups was noticed in the total scores at 3 years. Elimination of CUI is important for improving postoperative sexual function [29, 32]. In our study, questions regarding CUI or fear of incontinence restricting sexual activity showed significant improvement in both groups. Better objective cure is likely to explain the better outcome in the TVT group compared to the PAHG group [19]. There was no difference between the groups in turning sexually active or inactive postoperatively and as is to be expected, sexually inactive women were older and more often postmenopausal. We can’t, however, confirm that sexual inactivity was unrelated to SUI, as we didn’t ask the patients reasons for being or turning sexually inactive. De-novo dyspareunia and pain have been reported after mid-urethral sling surgery [7, 9], and dyspareunia is a common cause to seek management after synthetic mesh for SUI or pelvic organ prolapse [33]. In our study, PISQ-12 showed no increase in pain during sexual intercourse in either group after surgery, and there was no difference between the groups at 3 years. Incontinence-specific UDI-6 question for pain or discomfort and pain dimension of RAND-36 also showed no change from the baseline.

Urinary incontinence negatively impacts HRQoL [34, 35], also seen in our study with significantly lower baseline RAND-36 scores for physical functioning compared to the mean scores of the age-matched Finnish women [21]. In both groups with postoperative improvement the 3-year scores reached the level of age-matched Finnish women [21], with better scores in the TVT group. Improvement in social functioning was also seen at 3 years, with no difference between the groups. Worsening from the baseline was not detected in any of the dimensions of RAND-36 in either group. Our data are in accordance with previous studies reporting improved QoL after mid-urethral sling surgery [6] and PAHG [13–15].

This study provides unique midterm data on the impact of TVT and PAHG on QoL and sexual function. As a strength of our study, we included only women with primary SUI, whereas previous studies evaluating PAHG were conducted with women suffering from mixed urinary incontinence and/or previously failed anti-incontinence surgery [11, 14, 15]. We also used validated disease specific and general questionnaires to assess changes in QoL and sexuality. And finally, in our randomized clinical trial the drop-out rate was low.

There are also limitations to address. Our study on QoL and sexual function was a secondary analysis, and the power might not have been adequate to show a statistically significant difference in all end-points. As we studied only TVT and PAHG treatments, our findings cannot be extended to other mid-urethral slings or bulking agents. We didn’t request the reasons for sexual inactivity and therefore cannot determine whether the reason for inactivity was SUI related. However, no significant increase or decrease in sexuality was seen after 3 years. And finally, we had a rather high number of women who crossed over to the other treatment. However, there were no differences when compared to the results in treatment received or intention-to-treat.

## Conclusions

Our study shows that in women with primary SUI, both TVT and PAHG treatments improved HRQoL and incontinence-related sexual dysfunction at 3 years, but TVT patients suffer from less distress when comparing the groups.

## References

[1] Haylen BT, de Ridder D, Freeman RM (2010). An International Urogynecological Association (IUGA)/International Continence Society (ICS) joint report on the terminology for female pelvic floor dysfunction. Int Urogynecol J.

[2] Botlero R, Urquhart DM, Davis SR, Bell RJ (2008). Prevalence and incidence of urinary incontinence in women: review of the literature and investigation of methodological issues. Int J Urol.

[3] Ragins AI, Shan J, Thom DH, Subak LL, Brown JS, Van Den Eeden SK (2008). Effects of urinary incontinence, comorbidity and race on quality of life outcomes in wome. J Urol.

[4] Duralde ER, Rowen TS (2017). Urinary incontinence and associated female sexual dysfunction. Sex Med Rev.

[5] Dumoulin C, Cacciari LP, Hay-Smith EJC. Pelvic floor muscle training versus no treatment, or inactive control treatments, for urinary incontinence in women. Cochrane Database Syst Rev. 2018;10(10):CD005654. 10.1002/14651858.CD005654.pub4. 10.1002/14651858.CD005654.pub4 PMC6516955 30288727

[6] Ford AA, Rogerson L, Cody JD, Aluko P, Ogah JA. Mid-urethral sling operations for stress urinary incontinence in women. Cochrane Database Syst Rev. 2017;7:CD006375. 10.1002/14651858.CD006375.pub4. 10.1002/14651858.CD006375.pub4 PMC6483329 28756647

[7] Mengerink BB, Van Leijsen SAL, Vierhout ME (2016). The impact of midurethral sling surgery on sexual activity and function in women with stress urinary incontinence. J Sex Med.

[8] Glass Clark SM, Huang Q, Sima AP, Siff LN (2020). Effect of surgery for stress incontinence on female sexual function. Obstet Gynecol.

[9] Blaivas JG, Purohit RS, Benedon MS (2015). Safety considerations for synthetic sling surgery. Nat Rev Urol.

[10] Berger AA, Tan-Kim J, Menefee SA (2021). The impact of the 2011 US Food and Drug Administration transvaginal mesh communication on utilization of synthetic mid-urethral sling procedures. Int Urogynecol J.

[11] Mouritsen L, Lose G, Møller-Bek K (2014). Long-term follow-up after urethral injection with polyacrylamide hydrogel for female stress incontinence. Acta Obstet Gynecol Scand.

[12] Siddiqui ZA, Abboudi H, Crawford R, Shah S (2017). Intraurethral bulking agents for the management of female stress urinary incontinence: a systematic review. Int Urogynecol J.

[13] Maggiore ULR, Alessandri F, Medica M, Gabelli M, Venturini PL, Ferrero S. Periurethral injection of polyacrylamide hydrogel for the treatment of stress urinary incontinence: the impact on female sexual function. J Sex Med. 2012;9(12):3255–63. 10.1111/j.1743-6109.2012.02955.x. 10.1111/j.1743-6109.2012.02955.x 23206347

[14] Lose G, Sørensen HC, Axelsen SM, Falconer C, Lobodasch K, Safwat T (2010). An open multicenter study of polyacrylamide hydrogel (Bulkamid®) for female stress and mixed urinary incontinence. Int Urogynecol J.

[15] Toozs-Hobson P, Al-Singary W, Fynes M, Tegerstedt G, Lose G (2012). Two-year follow-up of an open-label multicenter study of polyacrylamide hydrogel (Bulkamid®) for female stress and stress-predominant mixed incontinence. Int Urogynecol J.

[16] Svenningsen R, Staff AC, Schiøtz HA, Western K, Kulseng-Hanssen S (2013). Long-term follow-up of the retropubic tension-free vaginal tape procedure. Int Urogynecol J.

[17] Ulmsten U, Henriksson L, Johnson P, Varhos G. An ambulatory surgical procedure under local anesthesia for treatment of female urinary incontinence. Int Urogynecol J Pelvic Floor Dysfunct. 1996;7(2):81–5. Discussion 85–6. http://www.ncbi.nlm.nih.gov/pubmed/8798092. Accessed October 26, 2018. 10.1007/BF01902378 8798092

[18] Itkonen Freitas A-M, Mentula M, Rahkola-Soisalo P, Tulokas S, Mikkola TS. Tension-free vaginal tape surgery versus polyacrylamide hydrogel injection for primary stress urinary incontinence: a randomized clinical trial. J Urol. 2020;203(2):372–8. 10.1097/JU.0000000000000517. 10.1097/JU.0000000000000517 31479396

[19] Itkonen Freitas A-M, Isaksson C, Rahkola-Soisalo P, Tulokas S, Mentula M, Mikkola TS (2022). Tension-free vaginal tape and polyacrylamide hydrogel injection for primary stress urinary incontinence: 3-year followup from a randomized clinical trial. J Urol.

[20] Mattsson NK, Nieminen K, Heikkinen A-M, et al. Validation of the short forms of the Pelvic Floor Distress Inventory (PFDI-20), Pelvic Floor Impact Questionnaire (PFIQ-7), and Pelvic Organ Prolapse/Urinary Incontinence Sexual Questionnaire (PISQ-12) in Finnish. Health Qual Life Outcomes. 2017;15(1):88. 10.1186/s12955-017-0648-2. 10.1186/s12955-017-0648-2 PMC5414223 28464936

[21] Aalto AM, Aro A, Teperi J. RAND-36 as measure of health-related quality of life: reliability, construct validity and reference value in Finnish general population. National Research and Development Centre for Welfare and Health (STAKES), Helsinki. 1999. http://urn.fi/URN:NBN:fi-fe201211089642.

[22] Maggiore ULR, Alessandri F, Medica M, Gabelli M, Venturini PL, Ferrero S. Outpatient periurethral injections of polyacrylamide hydrogel for the treatment of female stress urinary incontinence: effectiveness and safety. Arch Gynecol Obstet. 2013;288(1):131–7. 10.1007/s00404-013-2718-y. 10.1007/s00404-013-2718-y 23371485

[23] Heinonen P, Ala-Nissilä S, Räty R, Laurikainen E, Kiilholma P. Objective cure rates and patient satisfaction after the transobturator tape procedure during 6.5-year follow-up. J Minim Invasive Gynecol. 2013;20(1):73–8. 10.1016/J.JMIG.2012.09.007. 10.1016/j.jmig.2012.09.007 23312245

[24] Wai CY, Curto TM, Zyczynski HM (2013). Patient satisfaction after midurethral sling surgery for stress urinary incontinence. Obstet Gynecol.

[25] Hoe V, Yao HH, Gough K, O’Connell HE (2022). Long-term outcomes of polyacrylamide hydrogel treatment in women with stress urinary incontinence. BJU Int.

[26] Itkonen Freitas AM, Mikkola TS, Rahkola-Soisalo P, Tulokas S, Mentula M (2021). Quality of life and sexual function after TVT surgery versus Bulkamid injection for primary stress urinary incontinence: 1 year results from a randomized clinical trial. Int Urogynecol J.

[27] Gafni-Kane A, Zhou Y, Botros SM (2016). Predictive modeling and threshold scores for care seeking among women with urinary incontinence: The short forms of the Pelvic Floor Distress Inventory and Urogenital Distress Inventory. Neurourol Urodyn.

[28] Skorupska K, Grzybowska ME, Kubik-Komar A, Rechberger T, Miotla P. Identification of the Urogenital Distress Inventory-6 and the Incontinence Impact Questionnaire-7 cutoff scores in urinary incontinent women. Health Qual Life Outcomes. 2021;19(1). 10.1186/S12955-021-01721-Z. 10.1186/s12955-021-01721-z PMC7962285 33726776

[29] Fatton B, de Tayrac R, Costa P (2014). Stress urinary incontinence and LUTS in women—effects on sexual function. Nat Rev Urol.

[30] Cláudia Bicudo-Fürst M, Henrique P, Leite B, et al. Female sexual function following surgical treatment of stress urinary incontinence: systematic review and meta-analysis. Sex Med Rev. 2018;6(2):224–33. 10.1016/j.sxmr.2017.10.005. 10.1016/j.sxmr.2017.10.005 29289535

[31] Latul YP, Casteleijn FM, Zwolsman SE, Roovers J-PWR. Sexual function following treatment for stress urinary incontinence with bulk injection therapy and mid-urethral sling surgery. J Sex Med. 2022;19:1116–23. 10.1016/j.jsxm.2022.03.620. 10.1016/j.jsxm.2022.03.620 35568668

[32] Szell N, Komisaruk B, Goldstein SW, Qu X (Harvey), Shaw M, Goldstein I. A meta-analysis detailing overall sexual function and orgasmic function in women undergoing midurethral sling surgery for stress incontinence. Sex Med. 2017;5(2):e84–93. 10.1016/J.ESXM.2016.12.001. 10.1016/j.esxm.2016.12.001 PMC5440638 28363810

[33] Abbott S, Unger CA, Evans JM (2014). Evaluation and management of complications from synthetic mesh after pelvic reconstructive surgery: a multicenter study. Am J Obstet Gynecol.

[34] Krhut J, Gärtner M, Mokris J (2018). Effect of severity of urinary incontinence on quality of life in women. Neurourol Urodyn.

[35] Coyne KS, Kvasz M, Ireland AM, Milsom I, Kopp ZS, Chapple CR (2012). Urinary incontinence and its relationship to mental health and health-related quality of life in men and women in Sweden, the United Kingdom, and the United States. Eur Urol.

